# Supplementary material for: Combining Cluster Analysis of Air Pollution and Meteorological Data with Receptor Model Results for Ambient PM2.5 and PM10
Source: Int J Environ Res Public Health. 2020 Nov 15;17(22):8455. doi: 10.3390/ijerph17228455 (PMC7697898; doi:10.3390/ijerph17228455)
Supplement: Supplementary file 1 [file ijerph-17-08455-s001.zip › Supplementary_material_Jorquera_Villalobos_revised.docx]

Supplementary material for

Combining cluster analysis of air pollution and meteorological data with receptor model results for ambient PM_2.5_ and PM_10_

Héctor Jorquera ^1,2*^ and Ana María Villalobos ^3^

^1^ Departamento de Ingeniería Química y Bioprocesos, Pontificia Universidad Católica de Chile, Santiago, Chile; [jorquera@ing.puc.cl](mailto:jorquera@ing.puc.cl)

^2^ Centro de Desarrollo Urbano Sustentable; [jorquera@ing.puc.cl](mailto:jorquera@ing.puc.cl)

^3^ DICTUC S.A., Vicuña Mackenna 4860, Santiago, Chile; [anamariav.i@hotmail.com](mailto:anamariav.i@hotmail.com)

***** Correspondence: [jorquera@ing.puc.cl](mailto:jorquera@ing.puc.cl); Tel.: +56-22-354-4421 (H.J.) +56-22-354-4233 (A.V.)

**Table S1.** Emission inventory for Calama^1^, year 2016 [ton/year].

| Sector | PM_10_ | | PM_2.5_ | | SO_2_ | | NOx | | CO | | VOC | |  |
| --- | --- | --- | --- | --- | --- | --- | --- | --- | --- | --- | --- | --- | --- |
| Agriculture | | 0.5 | | 0.4 | | 0.01 | | 2.9 | | 1.8 | | 0.5 | |
| Airport | | 1.4 | | 1.4 | | 17.5 | | 253.25 | | 188.7 | | 0.0 | |
| Motor Vehicles | | 23.8 | | 22.1 | | 1.3 | | 502.47 | | 1665.6 | | 471.3 | |
| Off-road engines | | 5.9 | | 5.7 | | 0.2 | | 50.25 | | 37.6 | | 8.6 | |
| Construction | | 8.7 | | 0.9 | | 0.0 | | 0.00 | | 0.00 | | 0.0 | |
| Residential | | 7.2 | | 6.8 | | 1.5 | | 42.36 | | 113.1 | | 33.1 | |
| Road dust | | 344.8 | | 50.1 | | 0 | | 0 | | 0 | | 0 | |
| Total | | 392.2 | | 87.3 | | 20.44 | | 851.26 | | 2008 | | 523.4 | |

^1^ Adapted from: DICTUC, 2019. Antecedentes tecnicos para el PDA de Calama [WWW Document]. URL http://planesynormas.mma.gob.cl/archivos/2019/proyectos/25052019_dictuc26ab19.rar (accessed 9.29.20).


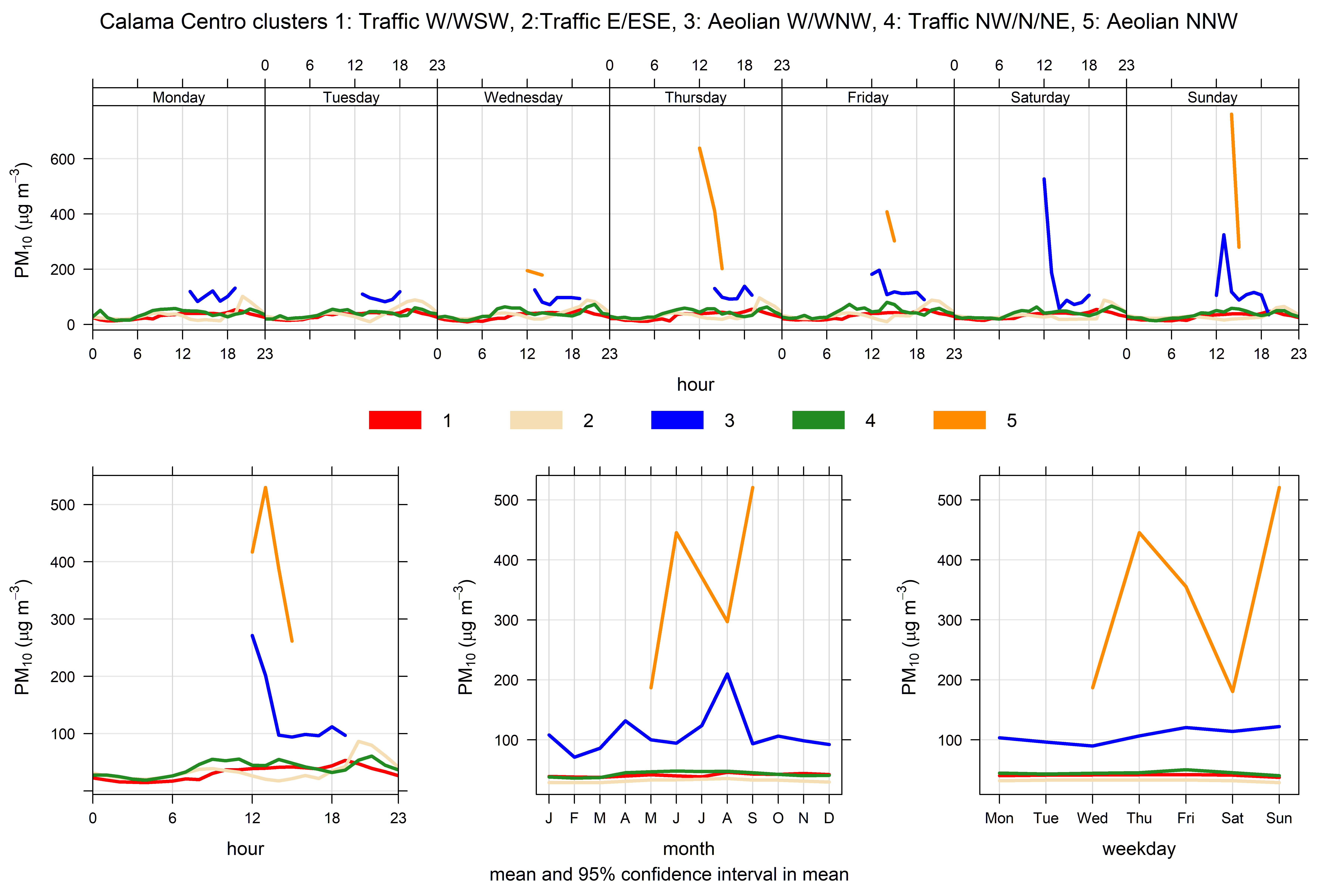


**Figure S1.** PM_10_ time variability results for a 5-cluster solution for Calama.


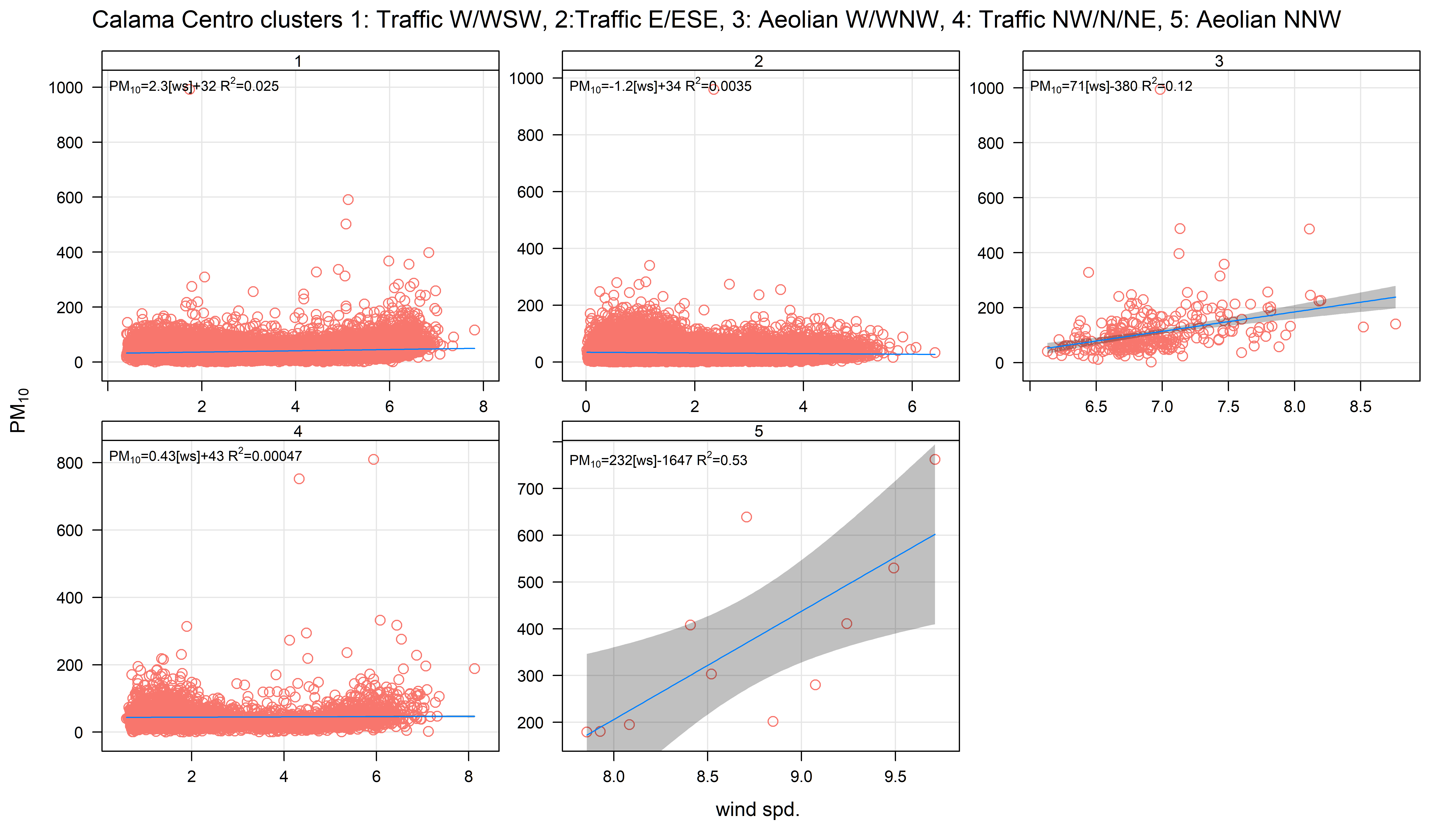


**Figure S2.** PM_10_ – wind speed scatter plot by cluster, for a 5-cluster solution for Calama.


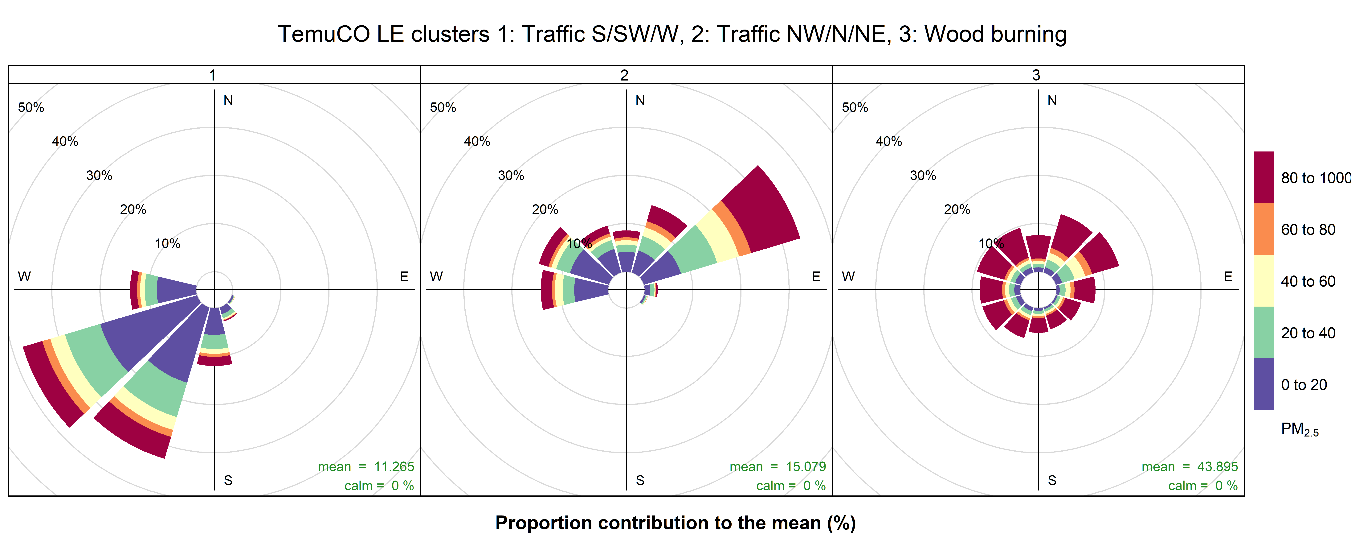


**Figure S3.** Pollution rose results by cluster, for a 3-cluster solution for Temuco.


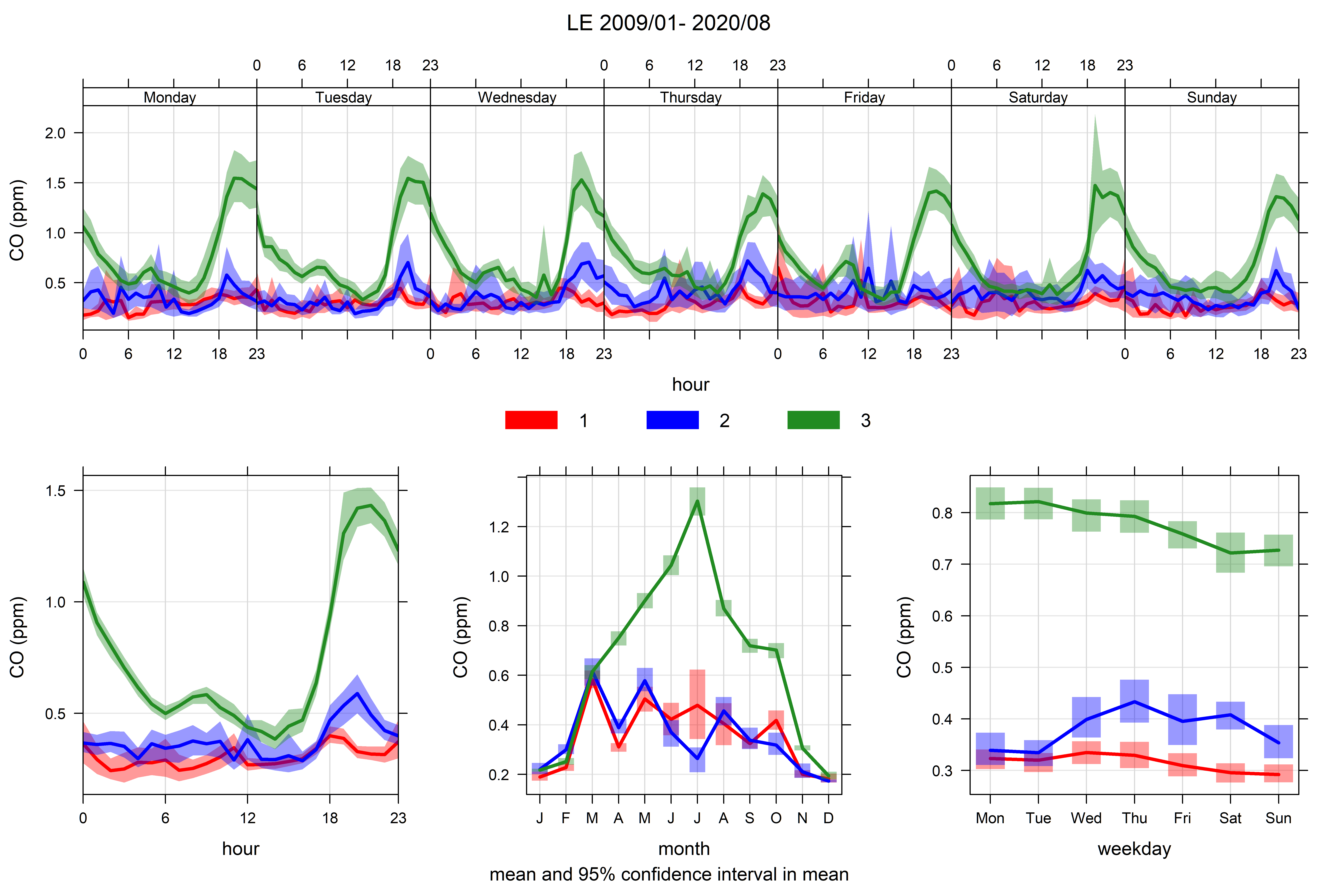


**Figure S4.** Time variability of CO by cluster, for a 3-cluster solution for Temuco.


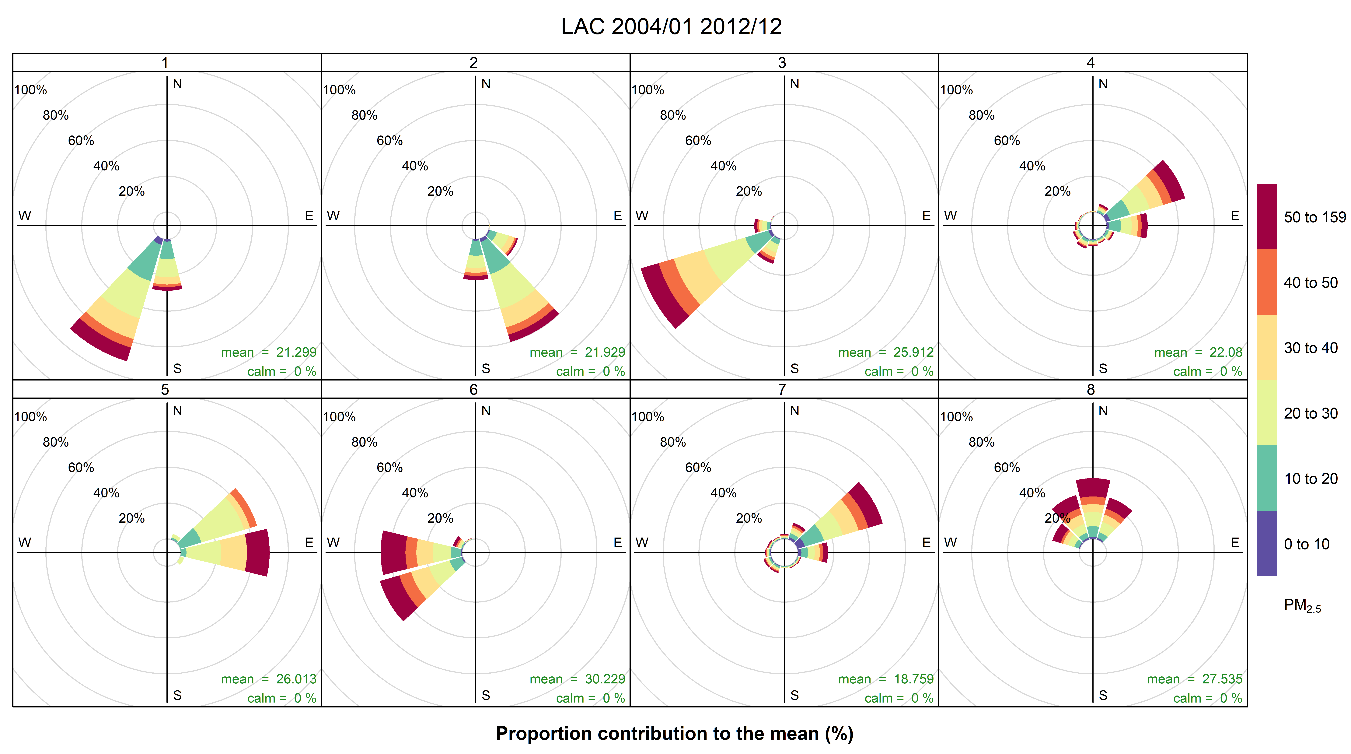


**Figure S5.** Pollution rose by cluster, for an 8-cluster solution for Santiago.


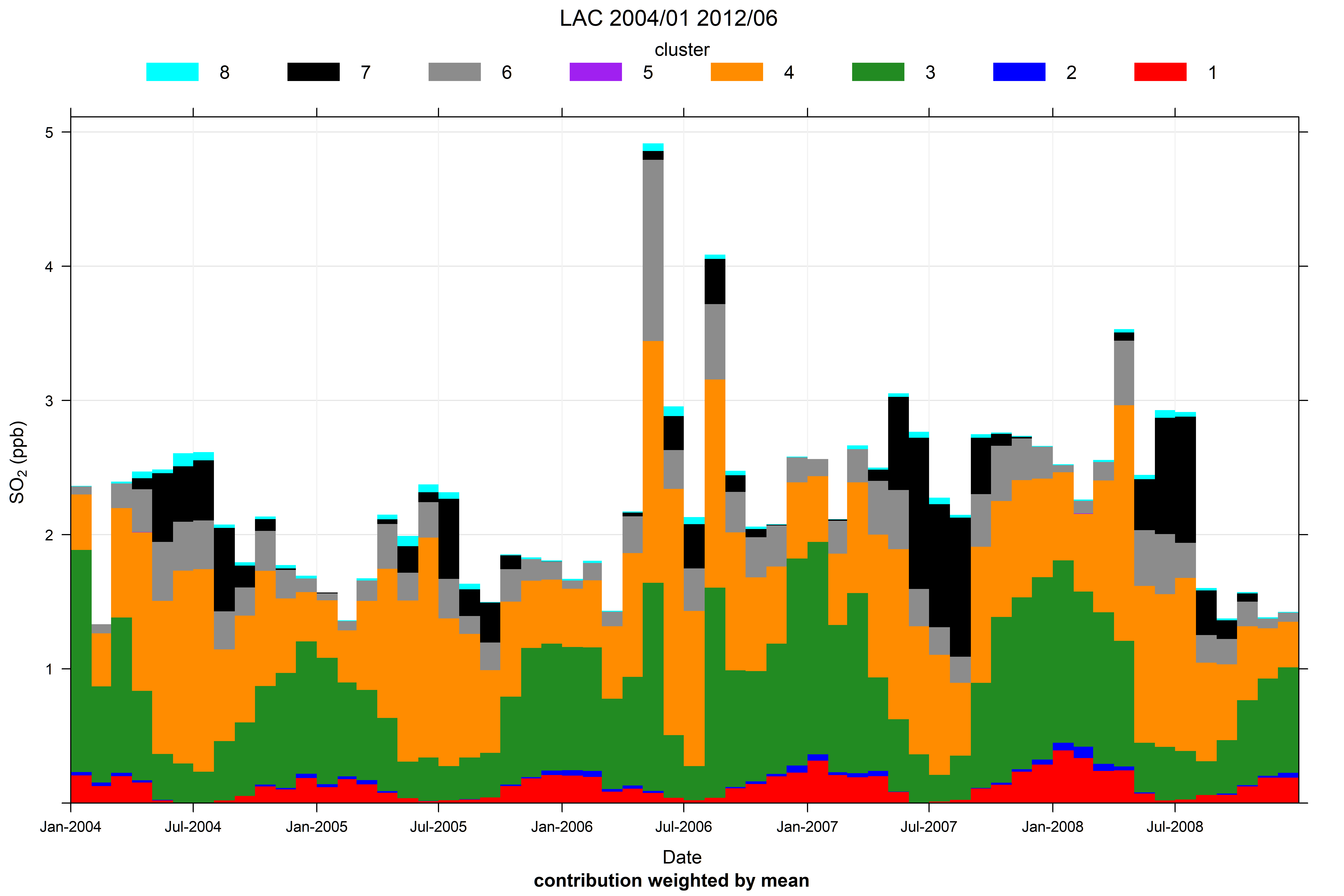


**Figure S6.** Source apportionment for SO_2_, for an 8-cluster solution for Santiago.
