# Supplementary material for: Combining Cluster Analysis of Air Pollution and Meteorological Data with Receptor Model Results for Ambient PM2.5 and PM10
Source: Int J Environ Res Public Health. 2020 Nov 15;17(22):8455. doi: 10.3390/ijerph17228455 (PMC7697898; doi:10.3390/ijerph17228455)
Supplement: Supplementary file 1 [file ijerph-17-08455-s001.zip › Supplementary_material_Jorquera_Villalobos_revised.pdf]

International Journal of *Environmental Research and Public Health*

MDPI

Supplementary material for

Combining cluster analysis of air pollution and meteorological data with receptor model results for ambient $PM_{2.5}$ and PM_{10}

Héctor Jorquera [1,2*] and Ana María Villalobos [3]

[1] Departamento de Ingeniería Química y Bioprocesos, Pontificia Universidad Católica de Chile, Santiago, Chile; jorquera@ing.puc.cl
[2] Centro de Desarrollo Urbano Sustentable; jorquera@ing.puc.cl
[3] DICTUC S.A., Vicuña Mackenna 4860, Santiago, Chile; anamariav.i@hotmail.com

* Correspondence: jorquera@ing.puc.cl; Tel.: +56-22-354-4421 (H.J.) +56-22-354-4233 (A.V.)

Table S1. Emission inventory for Calama[1], year 2016 [ton/year].

Sector	PM_{10}	$PM_{2.5}$	SO_2	NOx	CO	VOC
Agriculture	0.5	0.4	0.01	2.9	1.8	0.5
Airport	1.4	1.4	17.5	253.25	188.7	0.0
Motor Vehicles	23.8	22.1	1.3	502.47	1665.6	471.3
Off-road engines	5.9	5.7	0.2	50.25	37.6	8.6
Construction	8.7	0.9	0.0	0.00	0.00	0.0
Residential	7.2	6.8	1.5	42.36	113.1	33.1
Road dust	344.8	50.1	0	0	0	0
Total	392.2	87.3	20.44	851.26	2008	523.4

[1] Adapted from: DICTUC, 2019. Antecedentes tecnicos para el PDA de Calama [WWW Document]. URL http://planesynormas.mma.gob.cl/archivos/2019/proyectos/25052019_dictuc26ab19.rar (accessed 9.29.20).

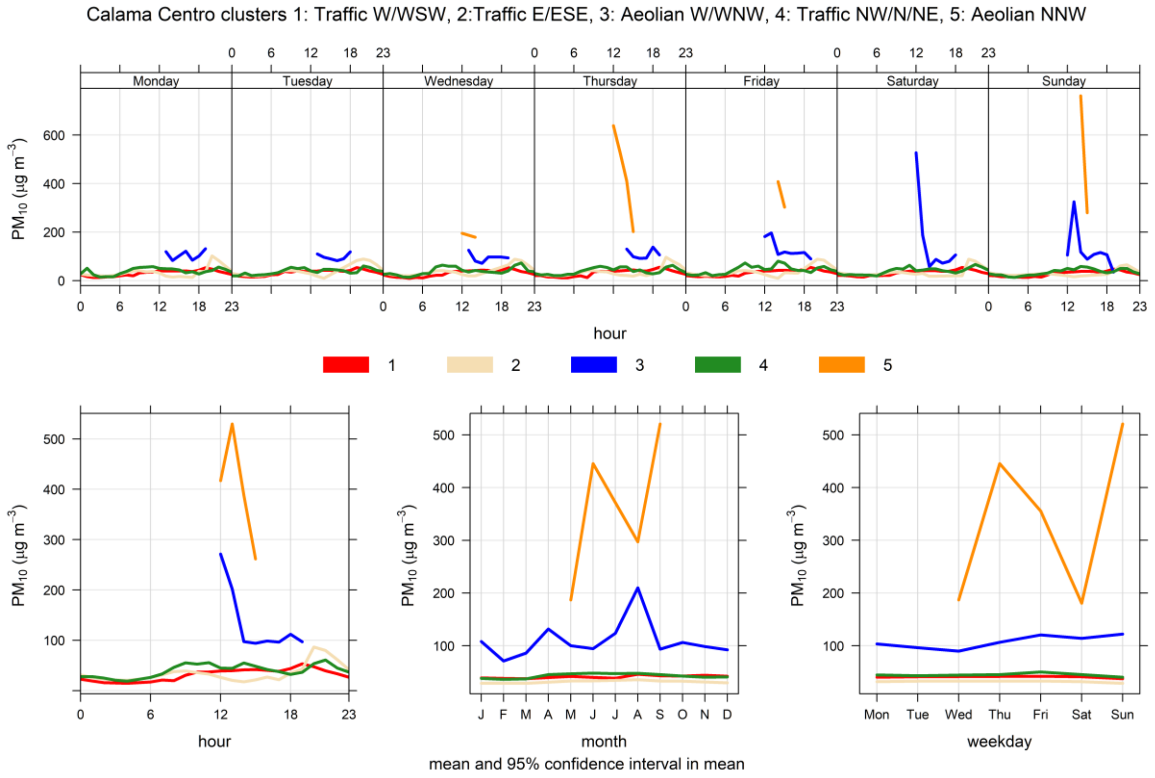

Figure S1. PM_{10} time variability results for a 5-cluster solution for Calama.

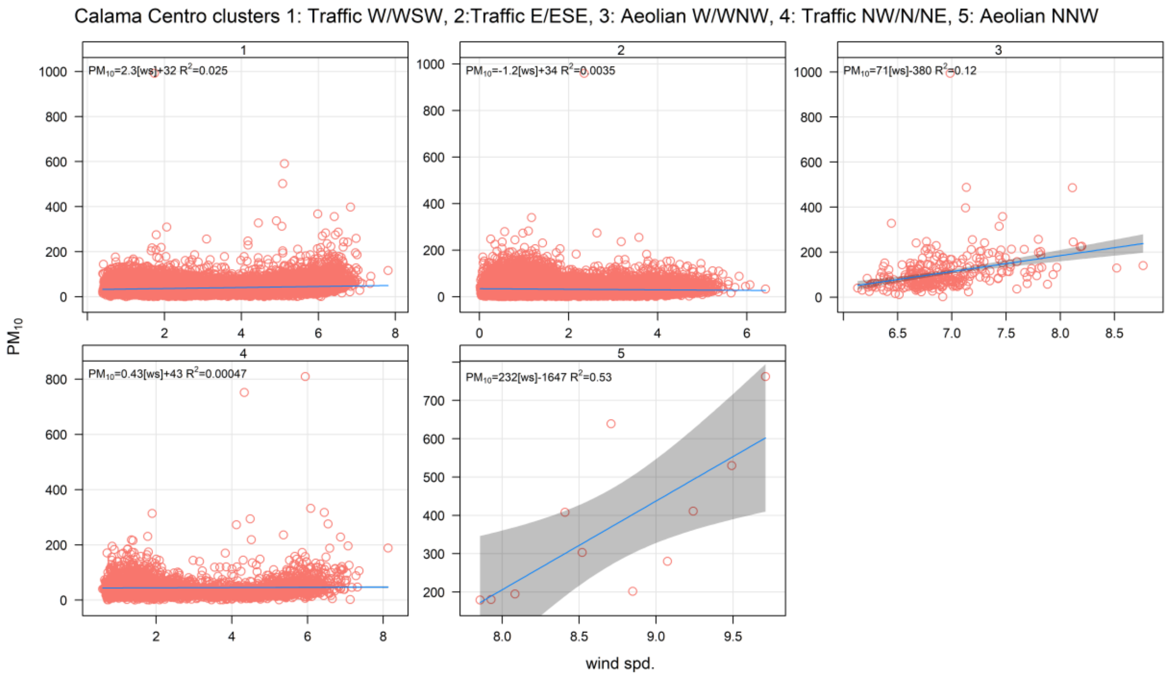

Figure S2. PM_{10} – wind speed scatter plot by cluster, for a 5-cluster solution for Calama.

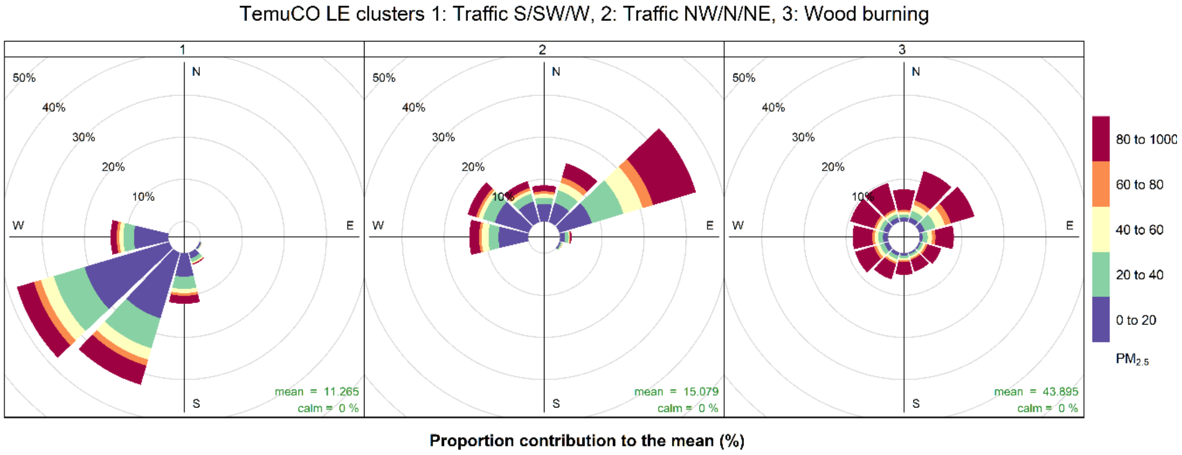

Figure S3. Pollution rose results by cluster, for a 3-cluster solution for Temuco.

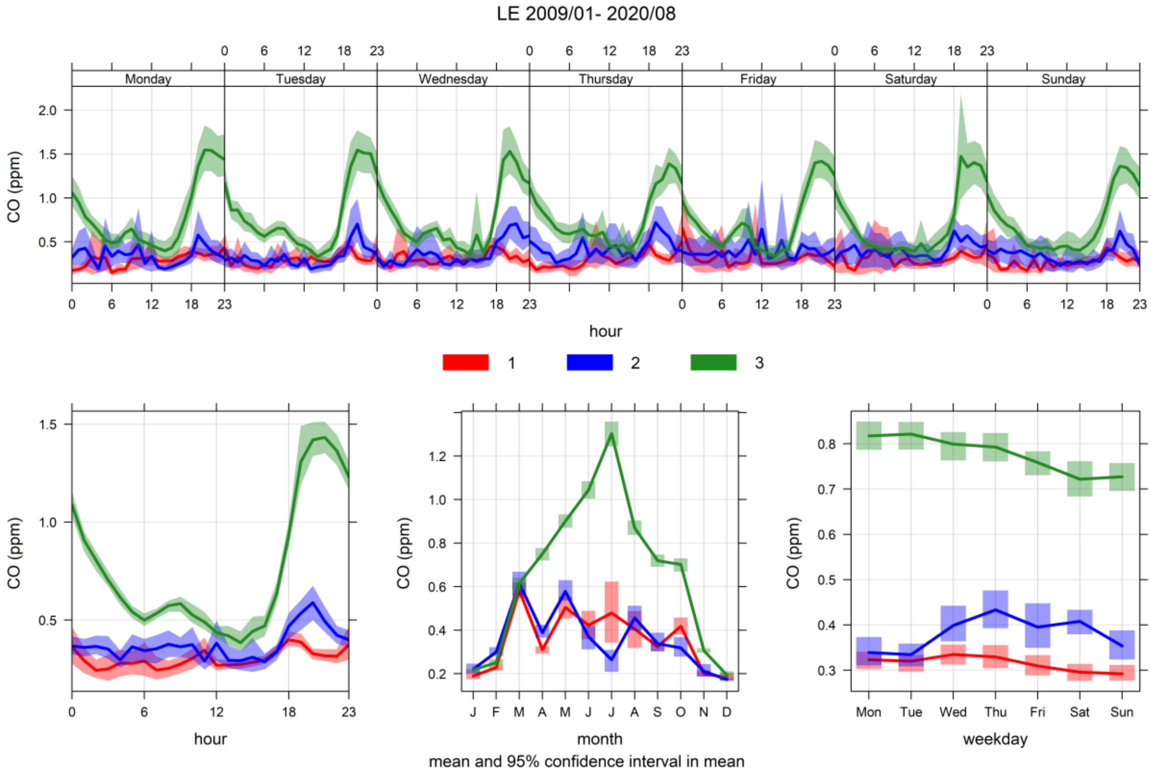

Figure S4. Time variability of CO by cluster, for a 3-cluster solution for Temuco.

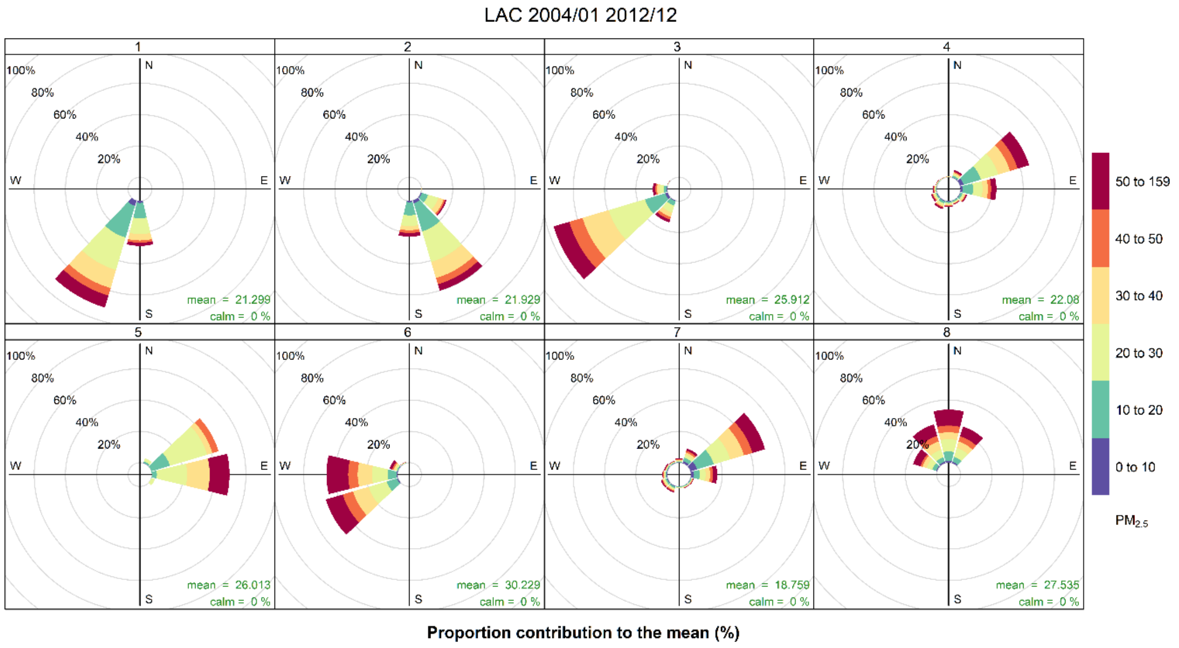

Figure S5. Pollution rose by cluster, for an 8-cluster solution for Santiago.

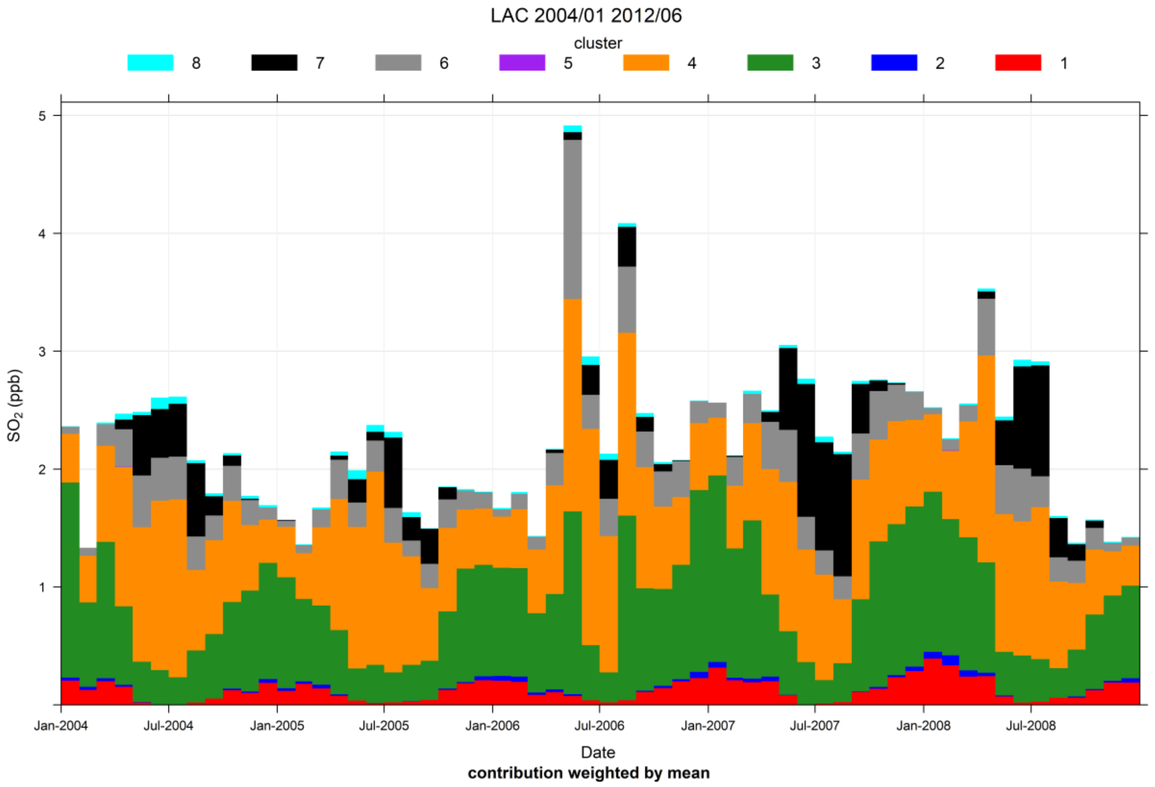

Figure S6. Source apportionment for SO_2, for an 8-cluster solution for Santiago.
